# Experience-dependent MeCP2 expression in the excitatory cells of mouse visual thalamus

**DOI:** 10.1371/journal.pone.0198268

**Published:** 2018-05-30

**Authors:** Yuki Yagasaki, Goichi Miyoshi, Mariko Miyata

**Affiliations:** 1 Department of Physiology I (Neurophysiology), Tokyo Women’s Medical University, School of Medicine, 8–1 Kawada-cho, Shinjuku-ku, Tokyo, Japan; 2 Division of Women Health Care Professionals and Researchers Support, Tokyo Women’s Medical University, 8–1 Kawada-cho, Shinjuku-ku, Tokyo, Japan; Virginia Tech Carilion Research Institute, UNITED STATES

## Abstract

Loss or gain of copy number of the gene encoding the transcription factor methyl-CpG-binding protein 2 (MeCP2) leads to neurodevelopmental disorders (Rett and MeCP2 duplication syndrome), indicating that precisely regulated MeCP2 expression during development is critical for mental health. Consistent with this idea, MeCP2 null mutants exhibit synaptic regression in the dorsal lateral geniculate nucleus (dLGN), the visual relay center in the thalamus, a phenotype resembling that of animals reared in the dark during the visual sensitive period. It remains unclear how MeCP2 expression is regulated during circuit formation and maturation, especially in excitatory and inhibitory populations of neurons. We found that, concomitant with the initiation of the dark-rearing sensitive period, MeCP2 protein levels were elevated in glutamatergic but not GABAergic neurons of the dLGN. Moreover, MeCP2 expression in glutamatergic populations was selectively reduced by dark-rearing. Therefore, we propose that visual experience–dependent MeCP2 induction in glutamatergic populations is essential for synaptic maturation within the dLGN.

## Introduction

Rett syndrome is a devastating neurodevelopmental disorder caused by mutations in methyl-CpG-binding protein 2 (MeCP2), an X-linked gene [1, 2]. MeCP2 mutations are lethal in males [3], whereas in females the symptoms of Rett syndrome vary depending on the mosaic state of X-chromosome inactivation in the human brain [1, 4]. To determine which brain circuit makes the greatest contribution to the Rett phenotype, MeCP2 has been ablated from specific cell populations in mouse models [5–13]. Interestingly, not only the loss but also gain of MeCP2 gene dosage affects mental health in both humans [14, 15] and mice [16–21], leading to the hypothesis that precise dosage regulation of MeCP2 is required for normal brain function [13, 19, 22].

During the postnatal developmental period, sensory activity–driven cues play important roles in shaping fully functional brain circuits [23–25]. Indeed, when the relevant sensory cues are not provided during the postnatal critical period, a brain circuit fails to fully refine and acquire computational ability [26–31]. In the case of the visual system, visual cues are initially processed by the retina; pass through the dorsal lateral geniculate nucleus (dLGN), the visual relay center in the thalamus; and ultimately arrive at the primary visual cortex (V1). In V1 neocortex, release of the neurotransmitter GABA from the inhibitory interneurons plays pivotal roles in regulating circuit plasticity and the critical period [28, 29, 32]. Neuronal MeCP2 expression in V1 becomes evident during the critical period, and removal of MeCP2 hastens the onset of the critical period [33]. Specifically, lack of MeCP2 expression in Pvalb (Parvalbumin)-positive GABAergic interneurons disrupts V1 ocular dominance plasticity [34]. Consistent with the idea that MeCP2 expression in GABAergic interneurons is involved in circuit maturation, MeCP2 loss-of-function in GABAergic cells is sufficient to induce a Rett-like phenotype in mice [5, 13]. However, the developmental timing of MeCP2 expression within the visual relay center of the thalamus, and its correlation to the visual critical period, have not been investigated.

Although the visual sensitive period also exists in the dLGN [35–37], little is known about the maturation mechanisms of this subcortical visual relay center, and in particular the roles of excitatory and inhibitory circuits. During early postnatal development, axonal fibers from retinal ganglion cells segregate into eye-specific projection domains and form abundant contacts onto relay neurons in the dLGN. Subsequently, these retinogeniculate synapses undergo refinement and strengthening before reaching maturity. Notably, when a mouse is dark-reared during the visual sensitive period (around P20-P34), the retinogeniculate synapses are remodeled to an immature-like state [35–37], indicating that visual experience is required for maintenance of mature synapses. Interestingly, similar immature synaptic phenotypes are found in the dLGN of normally reared MeCP2-null mutants [38], suggesting that MeCP2 plays essential roles in the development of retinogeniculate synapses. However, it remains unknown how MeCP2 expression is regulated by visual experience in both pre- and post-synaptic neurons, as well as in excitatory versus inhibitory neurons within the dLGN.

In this study, we first asked whether MeCP2 expression levels change during the course of development, specifically within the dLGN. Next, we tested if MeCP2 expression is differentially regulated between excitatory and inhibitory neurons. Finally, we addressed whether visual sensory cues differentially regulate MeCP2 expression between the excitatory and inhibitory cell populations.

## Materials and methods

### Animals

All experiments were approved by the Animal Care and Use Committee of Tokyo Women’s Medical University and performed according to institutional guidelines. Both sexes of C57BL/6 mice (P10-P50; Japan SLC Inc., Hamamatsu, Japan) and male MeCP2 knockout (KO) mice [B6.129P2(C)-MeCP2tm1.1Bird/J, Stock number; 003890, The Jackson Laboratory, Bar Harbor, ME] were used for experiments. The mice were housed under controlled temperature and humidity conditions, and had free access to food and water. Mice were reared under a 12:12 h light: dark cycle, except in dark-rearing experiments, during which mouse cages were placed in a light-tight box.

### Western blotting

Mice were decapitated under deep isoflurane anesthesia, and the brains were cooled in ice-cold phosphate buffered saline (PBS). Under a stereomicroscope (SZX12; Olympus, Tokyo, Japan), the cortex was peeled off to expose the thalamus. The dLGN was removed bilaterally by microdissection using micro-scissors, and dLGN samples pooled from three animals were immediately frozen in liquid nitrogen. Samples were lysed in sodium dodecyl sulfate (SDS) lysis buffer [2% SDS, 50 mM Tris-HCl (pH 6.8)] containing cocktails of protease inhibitors (CompleteMini; Roche, Basel, Switzerland) and phosphatase inhibitors (PhosSTOP, Roche). Protein concentration was quantified using a BCA Protein Assay Kit (Thermo Fisher Scientific, Waltham, MA, USA); the same amount of total protein was assayed in each western blot. Primary antibodies were used at the following dilutions: rabbit anti-MeCP2 (1:1000, 07–013, Merck Millipore, Darmstadt, Germany), mouse anti-β-actin (1:50000, A5441, Sigma-Aldrich, St. Louis, MO, USA). Band images were captured on an ImageQuant LAS4000 (GE Healthcare, Chicago, IL, USA). Immunoreactive bands around 75 kDa (MeCP2) and 42 kDa (β-actin) were analyzed using the ImageJ software. The level of MeCP2 protein in the dLGN was normalized against that of β-actin. Appropriate dLGN sampling was verified by performing Nissl staining on coronal sections of dissected thalamus (S1 Fig). Whole-brain lysate of MeCP2 KO mice (kindly provided by Dr. N. Kishi, Keio Univ., Tokyo, Japan) was prepared by the same procedure.

### Immunohistochemistry

Mice were deeply anesthetized with pentobarbital (50 mg/kg intraperitoneally) and perfused with freshly prepared 4% paraformaldehyde and 0.2% picric acid in 0.1 M phosphate buffer (pH 7.4). After perfusion, the brain and eyes were removed and post-fixed in the same fixative solution overnight, and then permeated with 10–30% sucrose in PBS. The samples were then frozen in O.C.T. compound (Sakura FineTechnical, Tokyo, Japan) and stored at -80°C until use. Fixed brain samples of MeCP2KO mice were prepared by the same procedure. Frozen samples were sectioned on a cryostat (Leica CM1850; Leica Microsystems, Nussloch, Germany) to a thickness of 20 μm. Sections were incubated with 10% normal donkey or goat serum to prevent nonspecific reactions, and then incubated with a mixture of primary antibodies: rabbit anti-MeCP2 antibody and goat anti-glutamic acid decarboxylase 1/2 antibody (GAD1/2, 1:200; GAD-Go-Af240, Frontier Institute, Hokkaido, Japan) or mouse anti-GAD2/1 antibody (1:10000, GC3108, Affinity Research Products, Exeter, UK). An appropriate Alexa Fluor–conjugated antibody (A-21082, A-10042, A-11037, and A-11029, Thermo Fisher Scientific) was used as the secondary antibody. Neurons were distinguished by a fluorescent Nissl stain using Neuro Trace 435/455(N-21479, Thermo Fisher Scientific) as previously described [39]. Fluorescence images were visualized by laser-scanning confocal microscopy (LSM710; Carl Zeiss, Jena, Germany) using a 10× (NA = 0.45) objective or an oil-immersion 63× objective (NA = 1.4) and acquired using the ZEN software (Carl Zeiss) with 1.58 μs pixel time and a 4-frame average at 512 × 512 pixels.

To quantitatively evaluate MeCP2 expression, fluorescence intensities in the dLGN or ventral posteromedial nucleus (VPM) were normalized against those in the external medullary lamina. Fluorescence intensities in ganglion cell layer or inner nuclear layer of the retina were normalized against those in the inner plexiform layer. To measure MeCP2 expression in GABAergic (GAD+, Nissl+) or glutamatergic (GAD-, Nissl+) neurons, MeCP2 fluorescence intensity in each cell was normalized against the background. To count the numbers of MeCP2 positive cells in GABAergic or glutamatergic neurons, triple stained images (MeCP2, GAD and Nissl) were analyzed using a cell counter plugin. All measurements were performed using ImageJ software. The average intensity in each animal was used for statistical analysis.

### Statistical analysis

Data are presented as means ± standard error of the mean. Most of the data did not have a normal distribution; therefore, we used nonparametric statistical analysis. The Steel–Dwass test was used for multiple comparisons, and the Wilcoxon test was used for two-group comparisons. Statistical tests were performed with the JMP Pro 13 software (SAS Institute Inc., Cary, NC, USA); P < 0.05 was taken to indicate a significant difference.

## Results

### MeCP2 expression in the visual thalamus increases during the visual sensitive period

**Fig 1:**
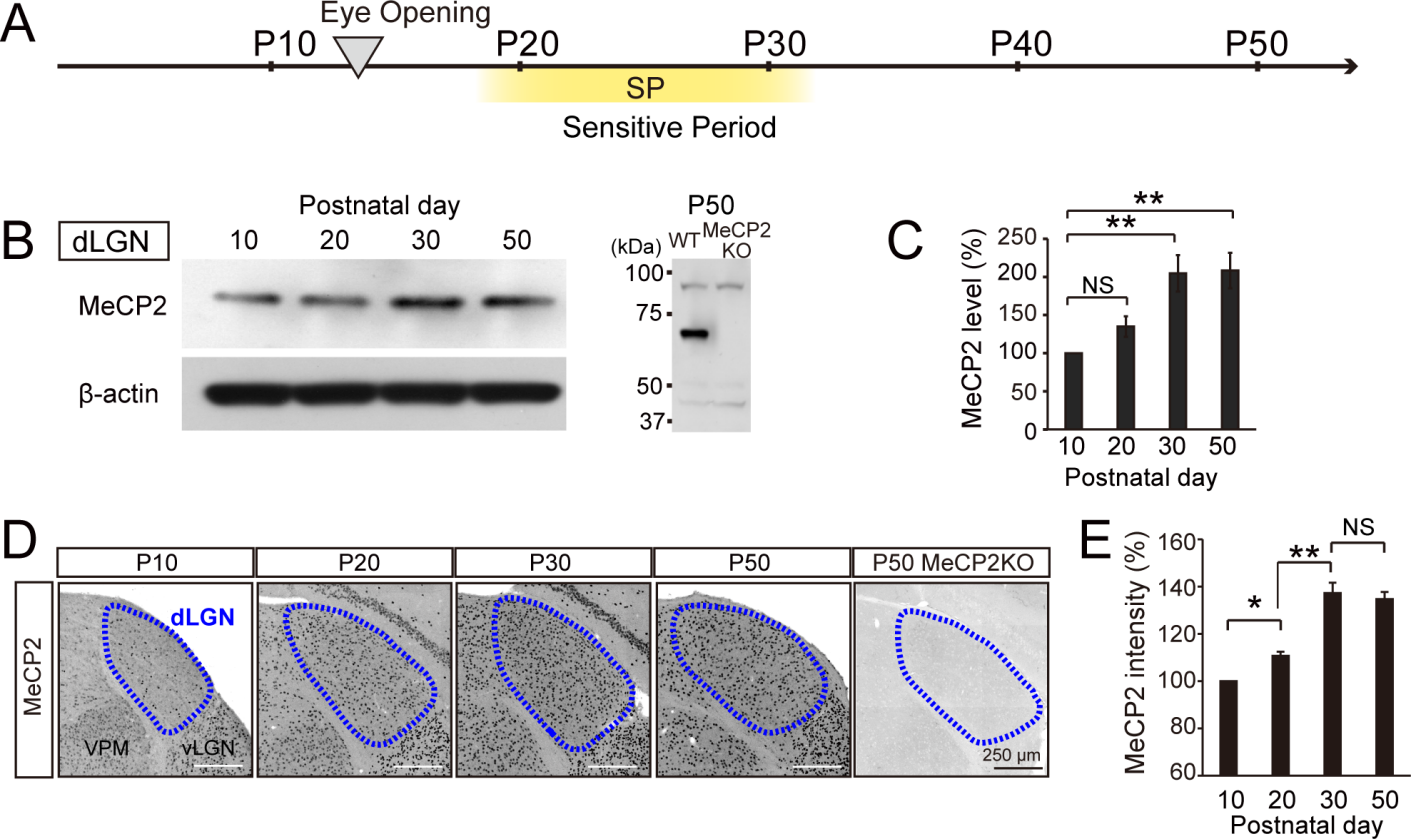
MeCP2 expression in the dLGN is elevated during the sensitive period of the visual system. **(A)** Developmental events involving the visual system. Reverse triangle: eye opening at P12–P14; SP, sensitive period. **(B)** Western blots for MeCP2 using dLGN lysate of wild-type (WT) mice obtained at P10, P20, P30, and P50 (left) or whole-brain lysate of MeCP2 KO mice (right) obtained at P50. **(C)** Quantification of MeCP2 expression level. The MeCP2 level at P10 was defined by 100%. **, P < 0.01. Statistical analysis for each developmental period was performed using the Steel-Dwass test. NS, not significant. n = 6 sample for 18 animals. **(D)** MeCP2 immunohistochemical staining in dLGN of WT mice at P10, P20, P30, and P50, and MeCP2 KO mice at P50. Blue dotted lines, dLGN. VPM: ventral posteromedial nucleus. vLGN: ventral lateral geniculate nucleus. Scale bar, 250 μm. **(E)** Quantification of MeCP2 fluorescence intensity in dLGN. MeCP2 intensity at P10 was defined as 100%. P20: 110.7% ± 1.7% vs. P30: 137.3% ± 4.3%, **P < 0.01. Statistical analysis for each developmental period was performed using the Steel-Dwass test. NS, not significant. P10: n = 24 sections for 7mice, P20: n = 26 sections for 8 mice, P30: n = 22 sections for 7 mice, P50: n = 36 sections for 10 mice.

Previously, MeCP2 protein expression was observed in the developing dLGN by western blot analysis [38]. Thus, we first investigated whether MeCP2 expression is developmentally regulated in the dLGN of the visual thalamus. We specifically dissected out the dLGN between P10 and P50 and carried out western blot analyses with antibodies raised against MeCP2 (Fig 1B). MeCP2 expression levels increased between P20 and P30 (Fig 1B and 1C), which coincided well with the visual sensitive period (Fig 1A) of the dLGN [35, 36]. We then analyzed developmental MeCP2 expression at the cellular levels and compared expression in the dLGN with that in the VPM of the somatosensory thalamus. Consistent with the western blot analyses, MeCP2 levels were selectively increased between P20 and P30 in the dLGN (Fig 1D and 1E) but not in the VPM (S1 Table). For both western blot and immunohistochemistry, we validated the specificity of MeCP2 antibodies using MeCP2-null animals (Fig 1B and 1D). Based on these findings, we conclude that the MeCP2 expression levels in the dLGN are specifically elevated during the visual sensitive period of the thalamic dLGN.

### MeCP2 up-regulation takes place selectively in excitatory populations of the dLGN

**Fig 2:**
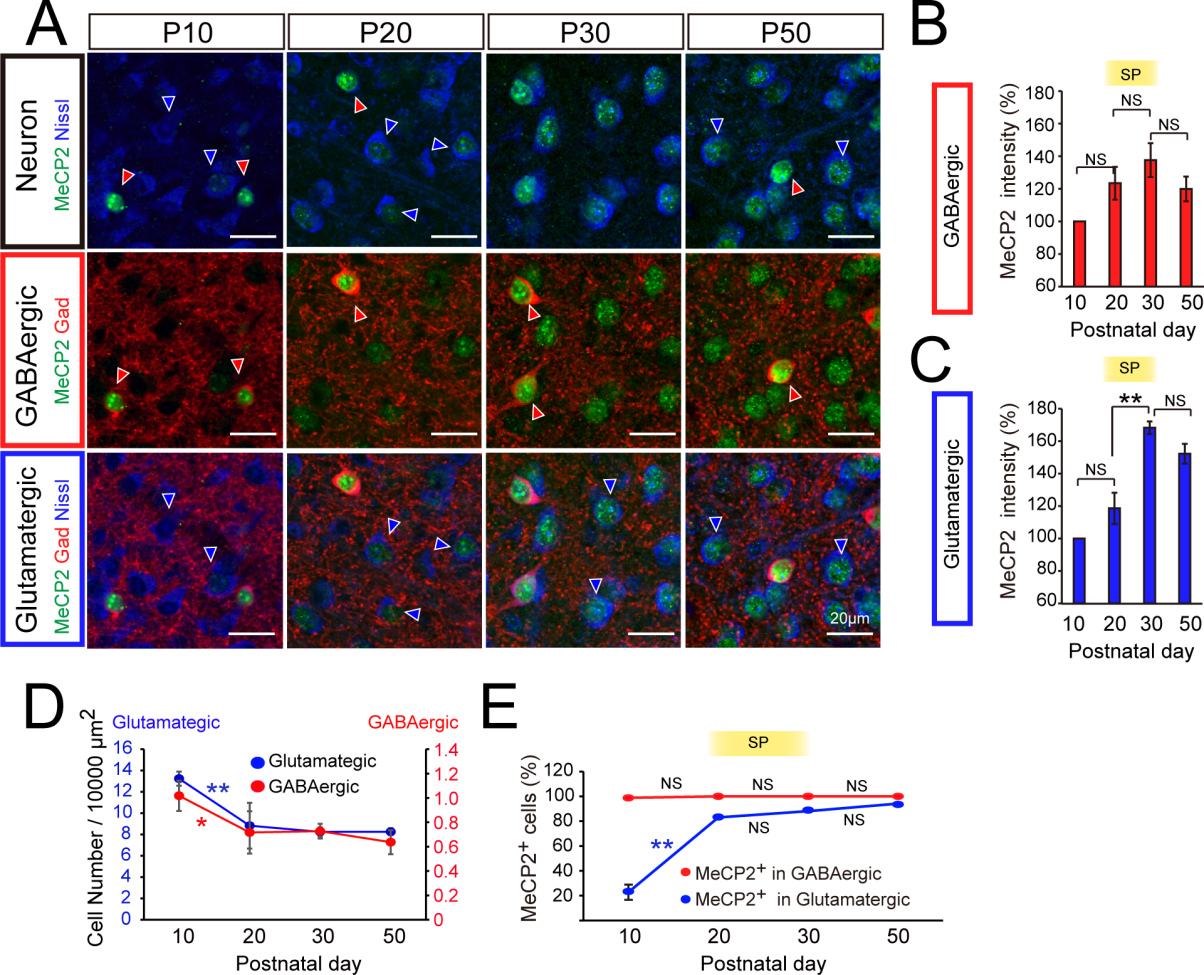
MeCP2 expression in excitatory versus inhibitory cells of the developing dLGN. **(A)** Identification of MeCP2-immunopositive cells (green) in the dLGN during development. Glutamatergic means GAD- / Nissl+ cells and GABAergic means GAD+ / Nissl+ cells. Red arrowheads, GABAergic neurons; blue arrowheads, glutamatergic neurons. Scale bar, 20 μm. **(B)** Changes in MeCP2-immunofluorescence intensity in GABAergic neurons during development. SP, sensitive period. P20: 123.4% ± 10.0% vs. P30: 137.6% ± 10.4%, P > 0.05. Statistical analysis for each developmental period was performed using the Steel-Dwass test. P10: 98 cells for 5 animals, P20: 56 cells for 6 animals. P30: 61 cells for 6 animals. P50: 44 cells for 5 animals. **(C)** Changes in MeCP2-immunofluorescence intensity in glutamatergic neurons during development. SP, sensitive period. P20: 118% ± 9.6% vs. P30: 168.3% ± 3.8%, **P < 0.01; Statistical analysis for each developmental period was performed using the Steel-Dwass test. NS, not significant. P10, 645 cells for 5 animals, P20: 738 cells for 6 animals. P30: 774 cells for 6 animals. P50: 463 cells for 5 animals. **(D)** The number of GABAergic and glutamatergic neuron in 10000 μm^2^ decreased between P10 and P20. GABAergic neuron, P10: 1.01 ± 0.12 vs P20: 0.72 ± 0.17, *P < 0.05. Glutamatergic neuron, P10: 13.23 ± 0.66 vs. P20: 8.82±2.1, **P < 0.01. Statistical analysis for each developmental period was performed using the Steel-Dwass test. N = 9 slides for 3 animals. **(E)** Proportion of MeCP2+ cells increased among glutamataargic neurons (blue), but not among GABAergic neurons (red), before entering the SP. Glutamatergic neurons, P10: 22.8% ± 6.2% vs. P20: 83.1% ± 2.2%, **P < 0.01; Statistical analysis for each developmental period was performed using the Steel-Dwass test. NS, not significant. N = 9 slides for 3 animals.

We next investigated whether MeCP2 up-regulation during the visual sensitive period differs between the excitatory relay neurons and inhibitory interneurons of the dLGN. To this end, we combined GABAergic (GAD) and neuronal (Nissl) markers to discriminate the glutamatergic (GAD-, Nissl+) and GABAergic (GAD+, Nissl+) neuronal populations (Fig 2A). Consistent with what we found in the dLGN as a whole (Fig 1), MeCP2 levels significantly increased during the visual sensitive period from P20 to P30 in the glutamatergic populations (Fig 2C, S2 Fig and S3 Fig). However, in the dLGN GABAergic cells, we observed no obvious increase in MeCP2 levels during this time window (Fig 2B, S2 Fig and S3 Fig). In addition to expression levels, we also analyzed the numbers of cells expressing MeCP2 in the dLGN. We found that MeCP2-positive cell numbers selectively increase in the glutamatergic but not in the GABAergic populations (Fig 2E and S2 Table). Consistent with previous reports, approximately 8% neurons in the dLGN were GAD positive (Fig 2D and S3 Table) [39, 40]. These data suggest that the MeCP2 expression is selectively induced in glutamatergic relay neurons of the dLGN during the visual sensitive period.

### Sensory experience during the visual sensitive period drives MeCP2 expression in the dLGN

**Fig 3:**
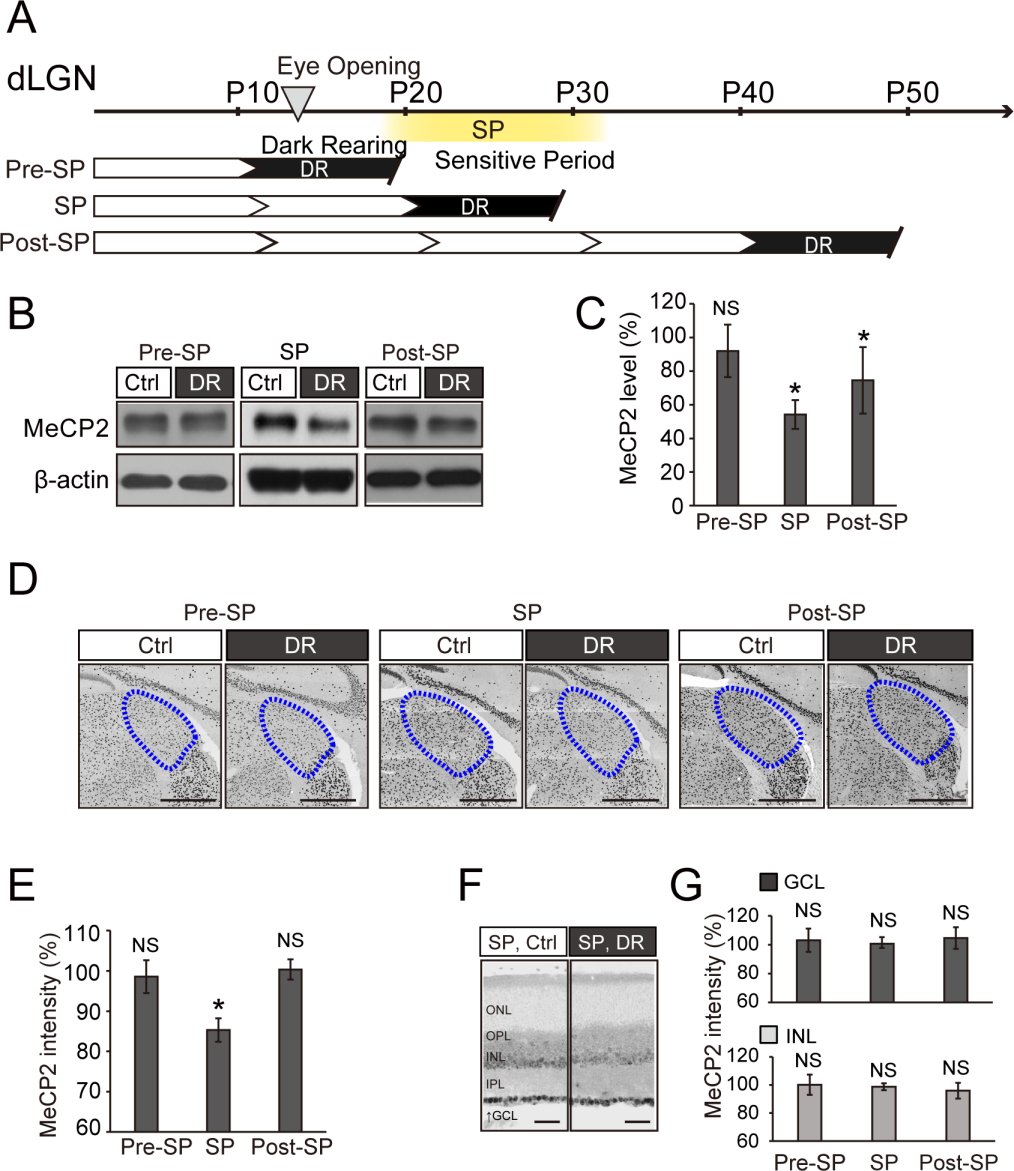
Dark-rearing during the visual sensitive period decreases MeCP2 expression within the dLGN. **(A)** Experimental design of dark-rearing and analysis. Dark-rearing (DR) was performed in each of the three developmental stages studied, and brains were collected at the end of each stage: Pre-SP, P11–P20; SP, P21–P30; and Post-SP, P41–P50. **(B)** Western blots for MeCP2 from dLGN of normally reared (control) and DR mice. **(C)** Quantification of MeCP2 expression levels. The MeCP2 level of each control sample was defined as 100%. Pre-SP: 92.0% ± 15.6% vs. normally reared control group, P > 0.05. SP: 54.2% ± 8.6% vs. control group, *P < 0.05. Post-SP: 74.5% ± 20.0% vs. control group, *P < 0.05; Wilcoxon test. NS, not significant. n = 6 samples for 18 animals. **(D)** Images of MeCP2 immunostaining in dLGN. Blue dotted lines, dLGN. Scale bar, 500 μm. **(E)** Quantification of MeCP2 immunofluorescence intensity in the dLGN. The MeCP2 level of each control sample was defined as 100%. SP: 85.3% ± 2.9% against control group, *P < 0.05; Statistical analysis for Ctrl vs DR was performed using the Wilcoxon test. NS, not significant. **(F)** Images of MeCP2 immunostaining in the retina from normally reared and DR mice. Scale bar, 50 μm. **(G)** Quantification of MeCP2 intensity in the GCL or INL in the retina. The MeCP2 level of each control sample was defined as 100%. ONL, outer nuclear layer; OPL, outer plexiform layer; INL, inner nuclear layer; IPL, inner plexiform layer; GCL, ganglion cell layer. Statistical analysis for Ctrl vs DR was performed using the Wilcoxon test. NS, not significant Pre-SP, SP, Post-SP: n = 16 sections from 4 mice.

Given that the timing of MeCP2 up-regulation coincides with the emergence of the visual sensitive period, we hypothesized that sensory experience induces MeCP2 expression in the dLGN. To directly test this possibility, we reared mice in the dark before (P11–P20), during (P21–P30), or after (P41–P50) the visual sensitive period, and then analyzed MeCP2 expression in the dLGN (experimental scheme: Fig 3A). Western blots on entire dLGN revealed that MeCP2 levels were significantly reduced when dark-rearing was performed during (SP), but not before (Pre-SP), the visual sensitive period (Fig 3B and 3C and S4 Fig). We also observed a slight decrease in MeCP2 expression levels even when dark-rearing was carried out after the visual sensitive period (Post-SP, Fig 3B and 3C and S4 Fig). However, when we analyzed MeCP2 expression at cellular resolution, we observed a specific decrease in the MeCP2 levels only if the dark-rearing was carried out during the visual sensitive period (Fig 3D). In stark contrast, dark-rearing did not affect MeCP2 expression in the visual pathway of the retina (Fig 3F and 3G). Therefore, visual experience is a critical driving force of MeCP2 expression within the dLGN.

### Sensory experience instructs excitatory dLGN populations to increase MeCP2 levels

**Fig 4:**
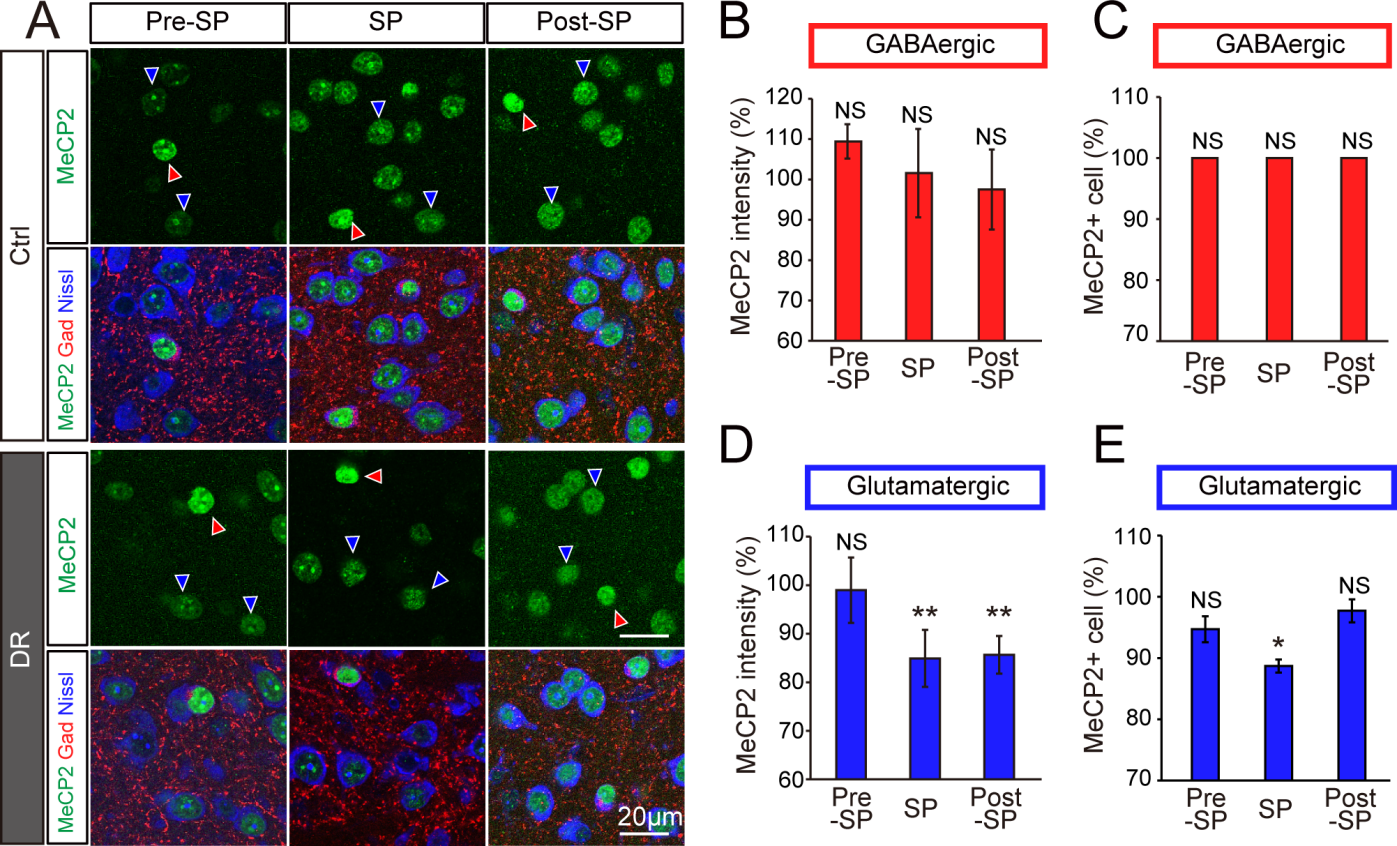
MeCP2 expression in glutamatergic dLGN neurons decreases upon dark-rearing. **(A)** Distribution of MeCP2-immunopositive cells (green) in the dLGN of normally reared control and dark-reared (DR) mice. Red arrowhead, GABAergic neurons (GAD+, Nissl+); blue arrowhead, glutamatergic neurons (GAD-, Nissl+). Scale bar, 20 μm. **(B)** Immunofluorescence intensity of MeCP2 signals in GABAergic neurons of the dLGN in DR mice at different developmental stages. **(C)** Proportion of MeCP2-positive cells among GABAergic neurons. NS, not significant. **(D) I**mmunofluorescence intensity of MeCP2 in glutamatergic neurons in the dLGN of DR mice. SP: 84.9% ± 5.9% vs. control group, **P < 0.01; Post-SP: 87.5% ± 3.9% vs. control group, **P < 0.01. Statistical analysis for Ctrl vs DR was performed using the Wilcoxon test. **(E)** Proportion of MeCP2-positive cells among glutamatergic neurons. SP: 88.7% ± 1.1% vs. normally reared control group, *P < 0.05. Statistical analysis for Ctrl vs DR was performed using the Wilcoxon test.

The dark-rearing experiment during the visual sensitive period decreased MeCP2 levels within cells of the dLGN (Fig 3D and 3E), suggesting that, during the visual sensitive period, sensory experience causes excitatory cells to increase MeCP2 expression. Alternatively, visual experience may help inhibitory cells to stably maintain MeCP2 levels specifically during the sensitive period. To distinguish between these possibilities, we again turned to molecular marker analyses to separately label glutamatergic (GAD-, Nissl+) and GABAergic (GAD+, Nissl+) cell types (Fig 4A and S5 Fig). Although we observed no obvious changes in MeCP2 expression levels or cell numbers within the GABAergic populations (Fig 4B and 4C, S3 Fig and S5 Fig), this was not the case in glutamatergic neurons (Fig 4D and 4E, S3 Fig and S5 Fig). Consistent with the outcome of whole-dLGN analyses (Fig 3B–3E), in the excitatory population, MeCP2 levels decreased when dark-rearing was carried out during or after the visual sensitive period (Fig 4D), and MeCP2-positive cell numbers were reduced when dark-rearing was carried out during the visual sensitive period (Fig 4E). These data strongly suggest that visual experience is required for glutamatergic dLGN cells to express MeCP2 at higher levels during the visual sensitive period. Based on these findings, we conclude that the sensory experience during the visual sensitive period instructs glutamatergic, but not GABAergic, neurons to increase MeCP2 expression levels in the dLGN.

## Discussion

The transcriptional regulator MeCP2 is the causal gene of Rett and MeCP2 duplication syndrome, and plays critical roles in neural circuit development, maturation, and function in multiple contexts. [1, 2, 41, 42]. In the visual sensory pathways, MeCP2 levels increase in the visual neocortex specifically during the critical period [33]. However, the regulation of MeCP2 expression in the visual relay center thalamus, and especially its cell-type specificity, has not been previously investigated.

**Fig 5:**
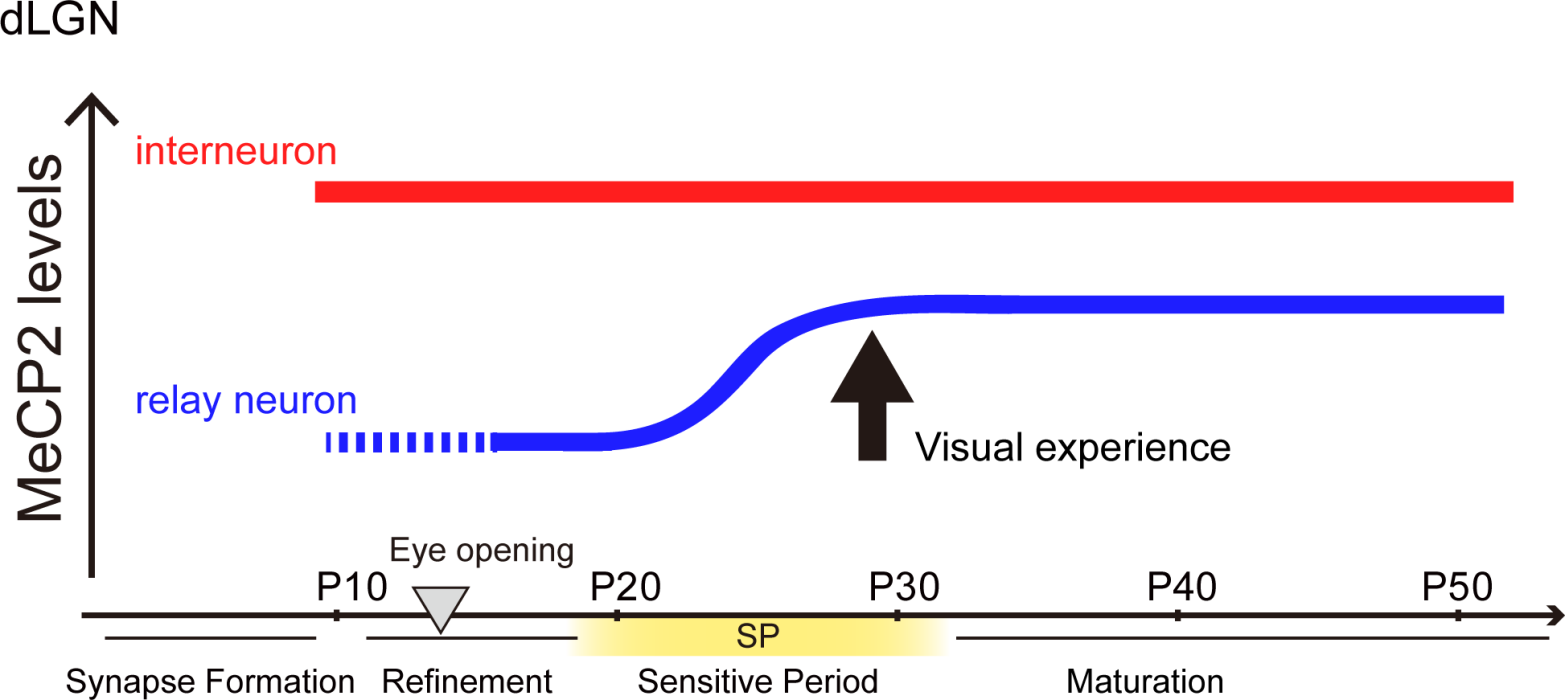
Postnatal MeCP2 expression in excitatory and inhibitory dLGN neurons. MeCP2 level is dramatically increased in glutamatergic neurons of the dLGN during the visual sensitive period. Visual deprivation (dark-rearing) during the sensitive period (SP) or post-SP prevents MeCP2 up-regulation in glutamatergic neurons. MeCP2 expression in GABAergic neurons is stably maintained, and is not affected by visual deprivation.

Here, we found that MeCP2 expression in the dLGN is up-regulated specifically during the sensitive period of visual sensory pathways, and that this takes place selectively in excitatory cell populations (summarized in Fig 5). The timing of MeCP2 up-regulation was not coincidental, as it was disrupted by visual sensory deprivation (dark-rearing) in excitatory cells of the dLGN. Our study strongly suggests that that sensory experience–driven induction of MeCP2 expression in excitatory cells is crucial to proper assembly of the thalamic vision relay center and establishment of a fully mature visual sensory brain circuit.

### Differential MeCP2 regulation between excitatory and inhibitory neuronal populations

We showed that MeCP2 expression is induced in the dLGN during the visual sensitive period in an experience-dependent manner. Interestingly, this event only occurred in the excitatory, but not in the inhibitory cell types. Contrary, MeCP2 expression was not sensitive to visual experience in either excitatory (GCL) or inhibitory (INL, including amacrine cells) cell types in the retina (Fig 3F and 3G). Furthermore, it is likely that MeCP2 plays little or no role in retinal development as no morphological change of retina is observed in MeCP2-null mutants [43] or Rett patients [44]. On the other hand, MeCP2 is an essential regulator of the neocortical plasticity [45, 46] and critical period [33, 34] of V1. In comparison with our measurements in thalamic dLGN, MeCP2 expression in the V1 cortex is low at P12, but is up-regulated upon eye opening at P15; at P30, after the critical period, expression reaches plateau levels and is maintained into adulthood [33]. Although the mechanisms responsible for its induction have not been well investigated, MeCP2 expression in GABAergic neurons in V1 is important for ocular dominance plasticity [33, 34]. Similar to what we observed in the visual thalamus, MeCP2 expression levels in the visual cortex may be regulated by visual experience–dependent neuronal activities, possibly in a cell type–specific manner.

### Roles of MeCP2 beyond circuit maturation

In MeCP2-null animals, eye-specific segregation and initial synapse formation of retinogeniculate projections are generally unaltered, but the subsequent visual experience–dependent maintenance phase is dramatically impaired, resembling the immature state [38]. This observation suggests that MeCP2 plays key roles in the maintenance of mature retinogeniculate synapses. *Is MeCP2 only transiently required during the maturation period*, *or is sustained expression necessary for the maintenance of a mature circuit*? As shown in Figs 3 and 4, we found that, even during the late maturation phase (P41–P50), MeCP2 expression is sensitive to lack of vision, although to a lesser extent than during the sensitive period (P21–P30). We recently showed that the metabotropic glutamate receptor (mGluR1) is necessary for maintenance of dLGN synapses [47]. Furthermore, to our surprise, activation of mGluR1 in the dLGN was sufficient to rescue the synaptic regression resulting from sensory deprivation during the visual sensitive period. It is reasonable to speculate that MeCP2 expression in the dLGN is regulated through the sensory experience–mGluR1 activation cascade in both the sensitive and mature periods. Consistent with this idea, mGluR1 is preferentially expressed in the excitatory relay neurons of the dLGN [48–50]. In preliminary experiments, we found that MeCP2 levels were elevated in the dLGN of mGluR1-null animals (S6 Fig). The MeCP2 level is significantly reduced in postmortem thalamus of Rett syndrome patients [51], implying that properly regulated MeCP2 expression within the thalamus is essential for mental health. Further studies are required to determine the potential roles of MeCP2 in mature thalamic circuits, as well as in excitatory and inhibitory neuronal populations.

## Conclusion

During the visual sensitive period, sensory experience selectively drives MeCP2 expression within the excitatory neurons of the thalamic visual relay center, the dLGN.

## Supporting information

S1 Fig Sampling of the dLGN. (A) To visualize the dLGN, Alexa488 conjugated CTB was injected into both sides of the retina. After 4 days, the cortex was peeled off under a fluorescent stereomicroscope (SZX12, Olympus) to confirm the location of the dLGN in the exposed thalamus. dLGN: dorsal lateral geniculate nucleus. SC: superior colliculus. Scale bar: 1mm. (B) For western blotting, dissection was performed without Alexa488 conjugated CTB injection. Appropriate dLGN sampling was verified by performing Nissl staining on coronal sections of the dissected thalamus. Red line: cutting area. Scale bar: 1mm.
(PDF)

S2 Fig MeCP2 and GAD immunohistochemical staining of the dLGN in WT mice during development. White dotted lines, dLGN. Scale bar 250 μm.
(PDF)

S3 Fig Distribution of MeCP2 intensity of each individual neuron. **(A)** Distribution of MeCP2 intensity of each individual Glutamatergic neuron is shown as a histogram. P10, 645 cells for 5 animals, Pre-SP, Ctrl: 738 cells for 6 animals. DR: 517 cells for 5 animals. SP, Ctrl: 774 cells for 6 animals. DR: 620 cells for 5 animals. Post-SP, Ctrl: 463 cells for 5 animals. DR: 401 cells for 5 animals. Statistical analysis for each developmental period was performed using the Steel-Dwass test. ^††^P<0.01. Statistical analysis for Ctrl vs DR was performed using the Wilcoxon test. **P<0.01.
**(B)** Distribution of MeCP2 intensity in each individual GABAergic neuron is shown as a histogram. P10 Ctrl: 98 cells for 5 animals, Pre-SP, Ctrl: 56 cells for 6 animals. DR: 52 cells for 5 animals. SP, Ctrl: 61 cells for 6 animals, DR: 66 cells for 5 animals. Post-SP: 44 cells for 5 animals. DR: 41 cells for 5 animals. Statistical analysis for Ctrl vs DR was performed using the Wilcoxon test.
(PDF)

S4 Fig Scanned original data for each signal in Fig 3B. (PDF)

S5 Fig Images of MeCP2 and Gad immunostaining of the dLGN of normally reared (ctrl) and dark reared (DR) mice. White dotted lines, dLGN. Scale bar, 500 μm.
(PDF)

S6 Fig Representative western blot of developmental changes in the MeCP2 protein level in the dLGN of the mGluR1 KO mouse. (PDF)

S1 Table Quantification of MeCP2 fluorescence intensity in the VPM. **(A)** Quantification of MeCP2 fluorescence intensity in the VPM during development. P10: n = 24 sections for 7 mice, P20: n = 26 sections for 8 mice, P30: n = 22 sections for 7 mice, P50: n = 36 sections for 10 mice. Statistical analysis for each developmental period was performed using the Steel-Dwass test. NS, not significant. **(B)** Quantification of MeCP2 immunofluorescence intensity in the VPM after dark rearing. Pre-SP, Ctrl: n = 26 sections for 8 mice, DR: n = 16 sections for 4 animals, SP, Ctrl: n = 22 sections for 7 mice, DR: n = 21 sections for 5 mice, Post-SP, Ctrl: n = 36 sections for 10 mice. DR: 19 sections for 5 mice. Statistical analysis for Ctrl vs DR was performed using the Wilcoxon test. NS, not significant.
(PDF)

S2 Table MeCP2+ cell number in GABAergic neurons (GAD+, Nissl+) and MeCP2+ cell number in glutamatergic(GAD-, Nissl+) neurons per 10000 μm2 area during development. (PDF)

S3 Table Proportion of GABAergic neurons (GAD+, Nissl+) in the dLGN neurons during development. (PDF)
