# Supplementary material for: Experience-dependent MeCP2 expression in the excitatory cells of mouse visual thalamus
Source: PLoS One. 2018 May 30;13(5):e0198268. doi: 10.1371/journal.pone.0198268 (PMC5976183; doi:10.1371/journal.pone.0198268)
Supplement: S1 Fig — (A) To visualize the dLGN, Alexa488 conjugated CTB was injected into both sides of the retina. After 4 days, the cortex was peeled off under a fluorescent stereomicroscope (SZX12, Olympus) to confirm the location of the dLGN in the exposed thalamus. dLGN: dorsal lateral geniculate nucleus. SC: superior colliculus. Scale bar: 1mm. (B) For western blotting, dissection was performed without Alexa488 conjugated CTB injection. Appropriate dLGN sampling was verified by performing Nissl staining on coronal sections of the dissected thalamus. Red line: cutting area. Scale bar: 1mm. (PDF) [file pone.0198268.s001.pdf]

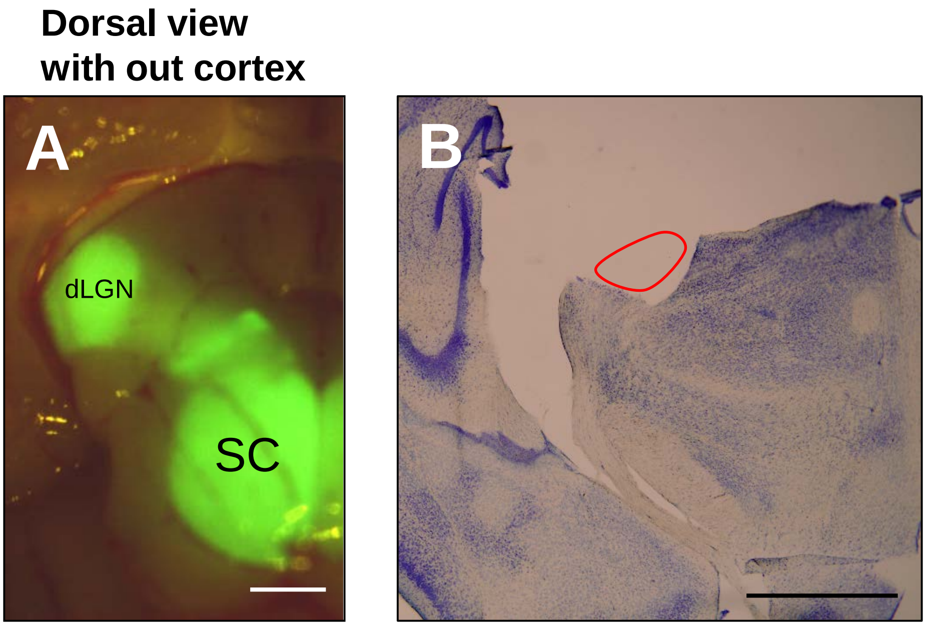

S1 Fig. Sampling of the dLGN

(A) To visualize the dLGN, Alexa488 conjugated CTB was injected into both sides of the retina. After 4 days, the cortex was peeled off under a fluorescent stereomicroscope (SZX12, Olympus) to confirm the location of the dLGN in the exposed thalamus. dLGN: dorsal lateral geniculate nucleus. SC: superior colliculus. Scale bar: 1mm

(B) For western blotting, dissection was performed without Alexa488 conjugated CTB injection. Appropriate dLGN sampling was verified by performing Nissl staining on coronal sections of the dissected thalamus. Red line: cutting area. Scale bar: 1mm.
