# Supplementary material for: Experience-dependent MeCP2 expression in the excitatory cells of mouse visual thalamus
Source: PLoS One. 2018 May 30;13(5):e0198268. doi: 10.1371/journal.pone.0198268 (PMC5976183; doi:10.1371/journal.pone.0198268)
Supplement: S2 Fig — White dotted lines, dLGN. Scale bar 250 μm. (PDF) [file pone.0198268.s002.pdf]

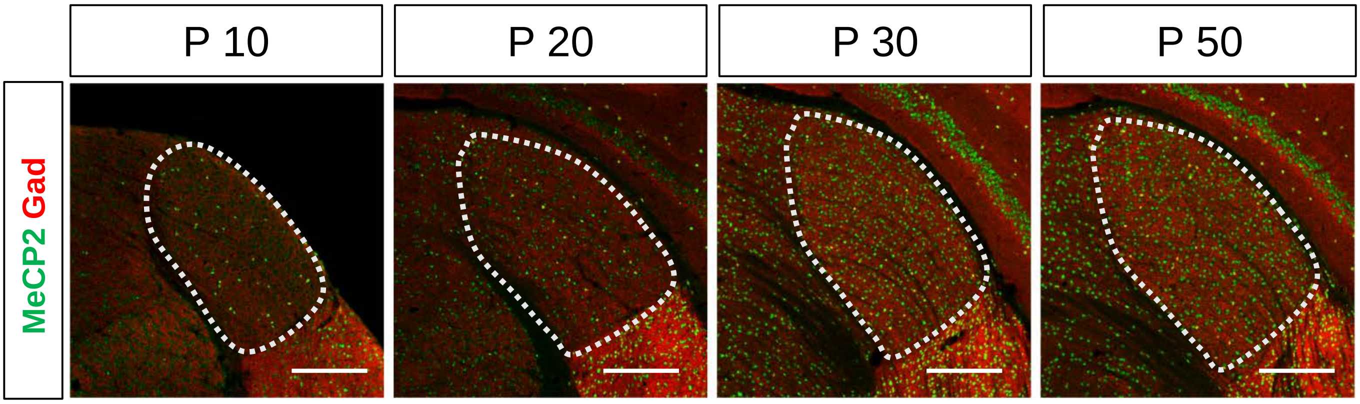

S2 Fig.

MeCP2 and GAD immunohistochemical staining of the dLGN in WT mice during development. White dotted lines, dLGN. Scale bar 250 µm.
