# Supplementary material for: Experience-dependent MeCP2 expression in the excitatory cells of mouse visual thalamus
Source: PLoS One. 2018 May 30;13(5):e0198268. doi: 10.1371/journal.pone.0198268 (PMC5976183; doi:10.1371/journal.pone.0198268)
Supplement: S3 Fig — (A) Distribution of MeCP2 intensity of each individual Glutamatergic neuron is shown as a histogram. P10, 645 cells for 5 animals, Pre-SP, Ctrl: 738 cells for 6 animals. DR: 517 cells for 5 animals. SP, Ctrl: 774 cells for 6 animals. DR: 620 cells for 5 animals. Post-SP, Ctrl: 463 cells for 5 animals. DR: 401 cells for 5 animals. Statistical analysis for each developmental period was performed using the Steel-Dwass test. ††P<0.01. Statistical analysis for Ctrl vs DR was performed using the Wilcoxon test. **P<0.01. (B) Distribution of MeCP2 intensity in each individual GABAergic neuron is shown as a histogram. P10 Ctrl: 98 cells for 5 animals, Pre-SP, Ctrl: 56 cells for 6 animals. DR: 52 cells for 5 animals. SP, Ctrl: 61 cells for 6 animals, DR: 66 cells for 5 animals. Post-SP: 44 cells for 5 animals. DR: 41 cells for 5 animals. Statistical analysis for Ctrl vs DR was performed using the Wilcoxon test. (PDF) [file pone.0198268.s003.pdf]

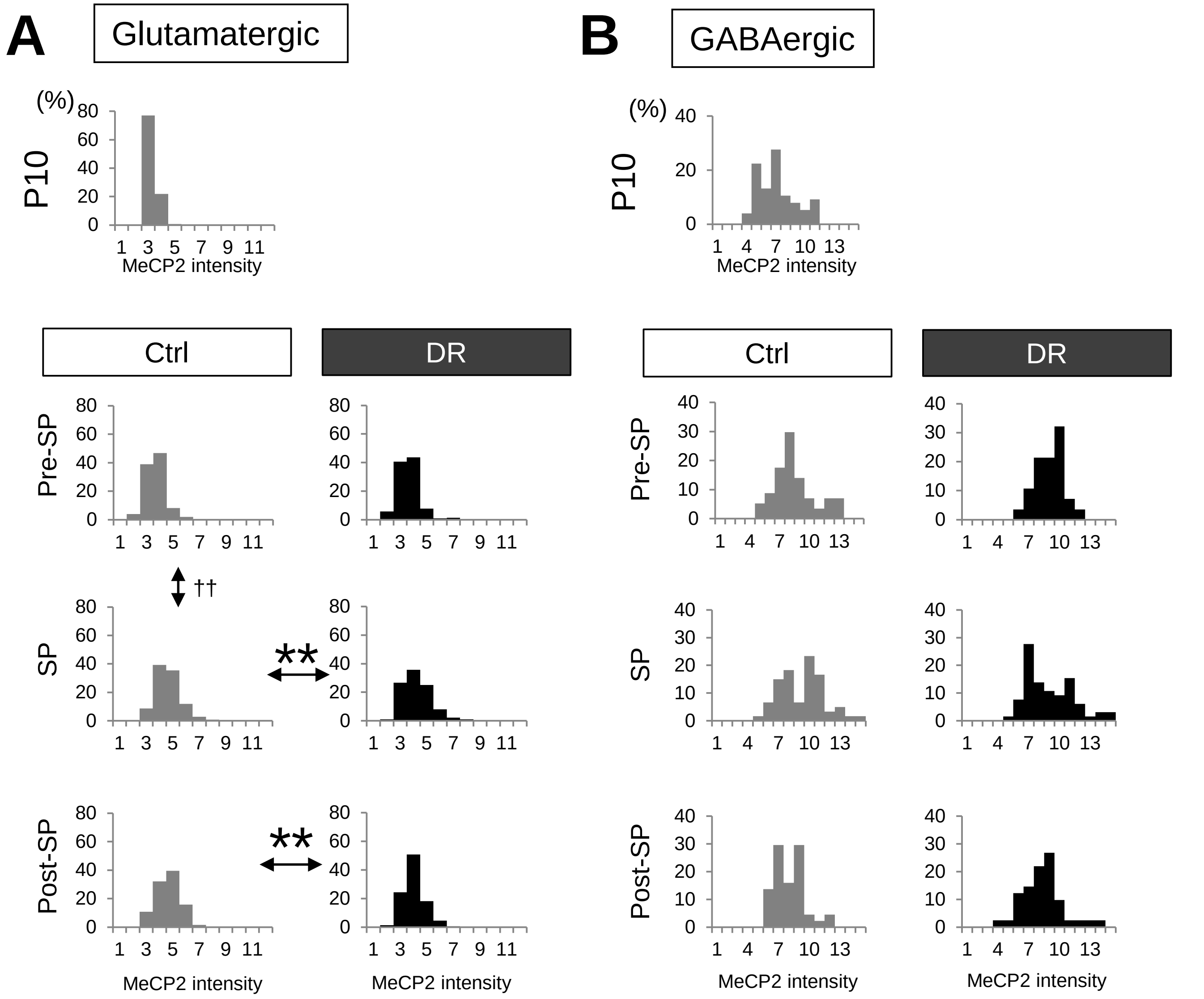


## S3 Fig.
## Distribution of MeCP2 intensity of each individual neuron

**(A)** Distribution of MeCP2 intensity of each individual Glutamatergic neuron is shown as a histogram. P10, 645 cells for 5 animals, Pre-SP, Ctrl: 738 cells for 6 animals. DR: 517 cells for 5 animals. SP, Ctrl : 774 cells for 6 animals. DR: 620 cells for 5 animals. Post-SP, Ctrl : 463 cells for 5 animals. DR: 401 cells for 5 animals. Statistical analysis for each developmental period was performed using the Steel-Dwass test. ††P<0.01. Statistical analysis for Ctrl vs DR was performed using the Wilcoxon test. **P<0.01.

**(B)** Distribution of MeCP2 intensity in each individual GABAergic neuron is shown as a histogram. P10 Ctrl: 98 cells for 5 animals, Pre-SP, Ctrl: 56 cells for 6 animals. DR: 52 cells for 5 animals. SP, Ctrl: 61 cells for 6 animals, DR: 66 cells for 5 animals. Post-SP: 44 cells for 5 animals. DR: 41 cells for 5 animals. Statistical analysis for Ctrl vs DR was performed using the Wilcoxon test.
