# Supplementary figures and images for: Experience-dependent MeCP2 expression in the excitatory cells of mouse visual thalamus
Source: PLoS One. 2018 May 30;13(5):e0198268. doi: 10.1371/journal.pone.0198268 (PMC5976183; doi:10.1371/journal.pone.0198268)

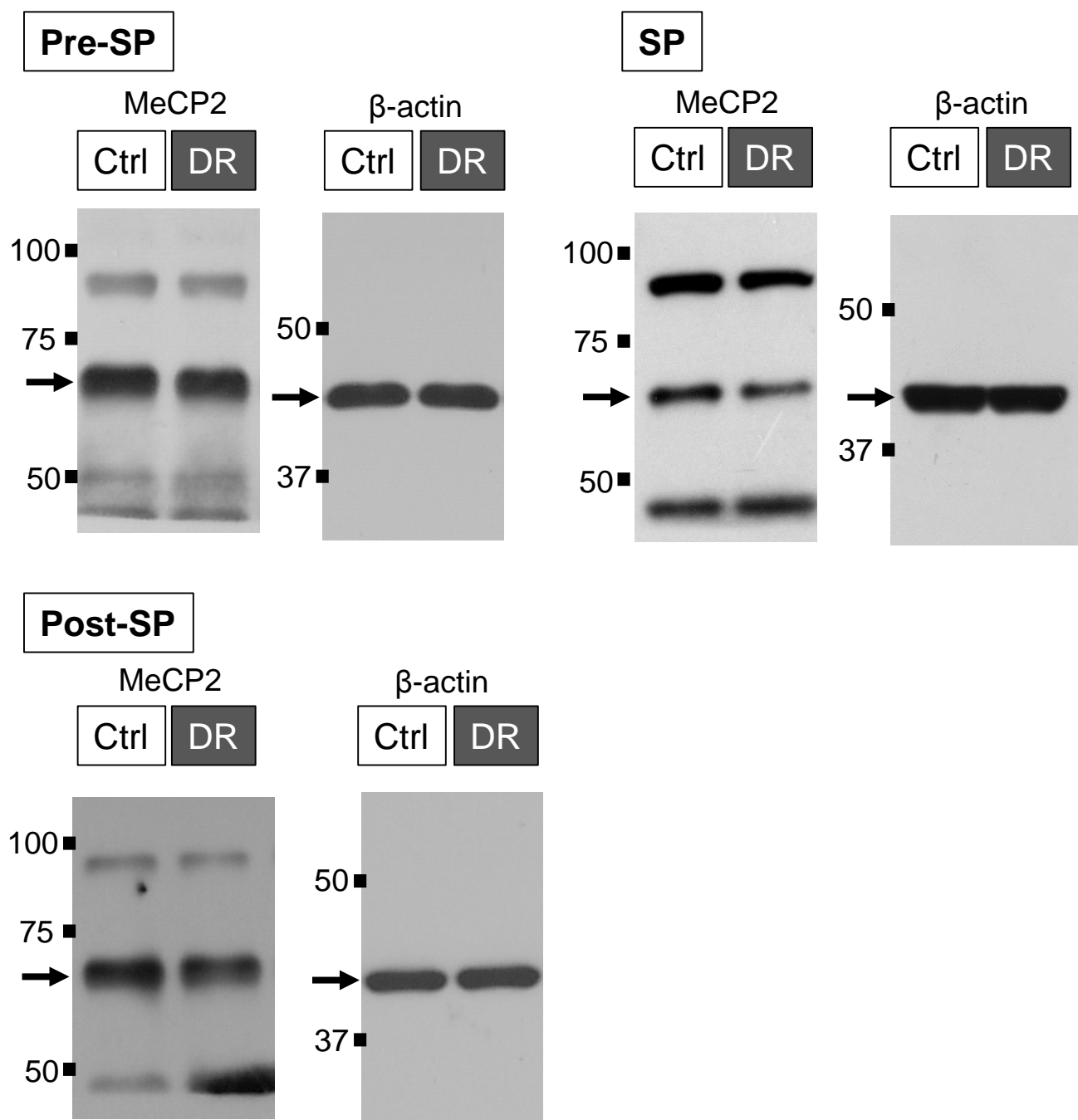

**S4 Fig.**  
Scanned original data for each signal in Fig 3B.

Supplement: S4 Fig — (PDF) [file pone.0198268.s004.pdf]
