# Supplementary material for: Experience-dependent MeCP2 expression in the excitatory cells of mouse visual thalamus
Source: PLoS One. 2018 May 30;13(5):e0198268. doi: 10.1371/journal.pone.0198268 (PMC5976183; doi:10.1371/journal.pone.0198268)
Supplement: S5 Fig — White dotted lines, dLGN. Scale bar, 500 μm. (PDF) [file pone.0198268.s005.pdf]

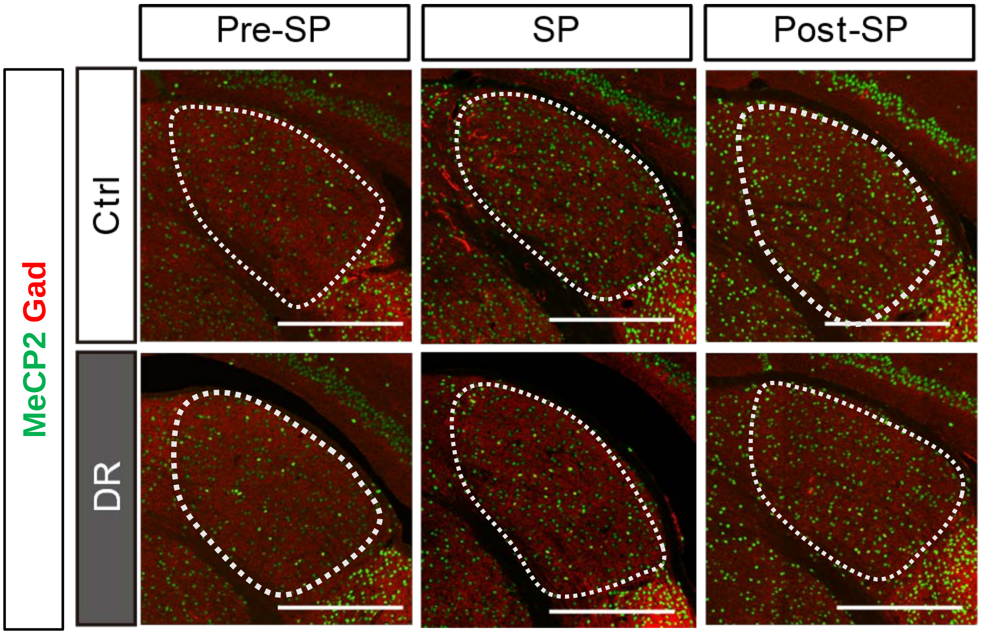

S5 Fig.

Images of MeCP2 and Gad immunostaining of the dLGN of normally reared (ctrl) and dark reared (DR) mice. White dotted lines, dLGN. Scale bar, 500 μm
