# Supplementary material for: Experience-dependent MeCP2 expression in the excitatory cells of mouse visual thalamus
Source: PLoS One. 2018 May 30;13(5):e0198268. doi: 10.1371/journal.pone.0198268 (PMC5976183; doi:10.1371/journal.pone.0198268)
Supplement: S6 Fig — (PDF) [file pone.0198268.s006.pdf]

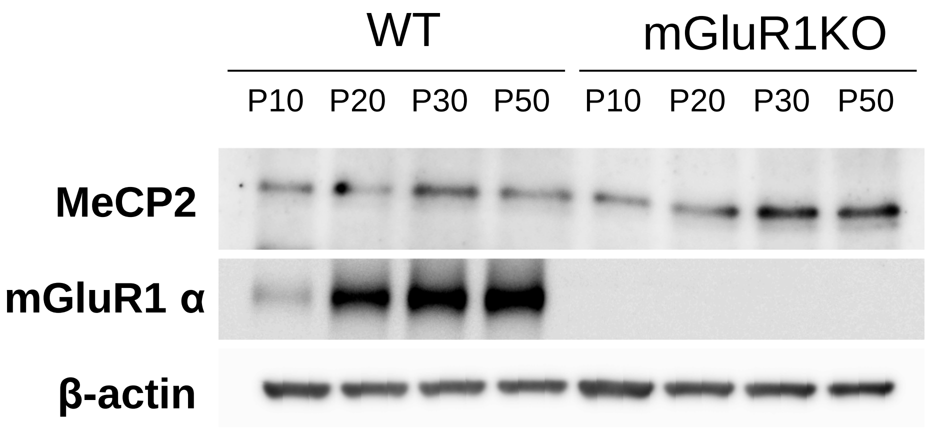

S6 Fig.

Representative western blot of developmental changes in the MeCP2 protein level in the dLGN of the mGluR1 KO mouse.
