# Supplementary material for: Experience-dependent MeCP2 expression in the excitatory cells of mouse visual thalamus
Source: PLoS One. 2018 May 30;13(5):e0198268. doi: 10.1371/journal.pone.0198268 (PMC5976183; doi:10.1371/journal.pone.0198268)
Supplement: S1 Table — (A) Quantification of MeCP2 fluorescence intensity in the VPM during development. P10: n = 24 sections for 7 mice, P20: n = 26 sections for 8 mice, P30: n = 22 sections for 7 mice, P50: n = 36 sections for 10 mice. Statistical analysis for each developmental period was performed using the Steel-Dwass test. NS, not significant. (B) Quantification of MeCP2 immunofluorescence intensity in the VPM after dark rearing. Pre-SP, Ctrl: n = 26 sections for 8 mice, DR: n = 16 sections for 4 animals, SP, Ctrl: n = 22 sections for 7 mice, DR: n = 21 sections for 5 mice, Post-SP, Ctrl: n = 36 sections for 10 mice. DR: 19 sections for 5 mice. Statistical analysis for Ctrl vs DR was performed using the Wilcoxon test. NS, not significant. (PDF) [file pone.0198268.s007.pdf]

A

	MeCP2 intensity (Average ± SEM)	Statistical Analysis Steel-Dwass test
P10	1.35 ± 0.07	P10 vs P20: NS
P20	1.37 ± 0.05	P20 vs P30: NS
P30	1.61 ± 0.08	P30 vs P50: NS
P50	1.63 ± 0.04	

B

	Ctrl MeCP2 intensity (Average ± SEM)	DR MeCP2 intensity (Average ± SEM)	Wilcoxon Test (Ctrl vs DR)
Pre-SP	1.37 ± 0.05	1.41 ± 0.03	NS
SP	1.61 ± 0.08	1.54 ± 0.03	NS
Post-SP	1.63 ± 0.04	1.65 ± 0.05	NS

S1 Table
Quantification of MeCP2 fluorescence intensity in the VPM

(A) Quantification of MeCP2 fluorescence intensity in the VPM during development. P10: n=24 sections for 7 mice, P20: n= 26 sections for 8 mice, P30: n=22 sections for 7 mice, P50: n=36 sections for 10 mice. . Statistical analysis for each developmental period was performed using the Steel-Dwass test. NS, not significant.

(B) Quantification of MeCP2 immunofluorescence intensity in the VPM after dark rearing. Pre-SP, Ctrl: n= 26 sections for 8 mice, DR: n=16 sections for 4 animals, SP, Ctrl: n=22 sections for 7 mice, DR: n=21 sections for 5 mice, Post-SP, Ctrl: n=36 sections for 10 mice. DR: 19 sections for 5 mice. Statistical analysis for Ctrl vs DR was performed using the Wilcoxon test. NS, not significant.
