# Supplementary material for: Experience-dependent MeCP2 expression in the excitatory cells of mouse visual thalamus
Source: PLoS One. 2018 May 30;13(5):e0198268. doi: 10.1371/journal.pone.0198268 (PMC5976183; doi:10.1371/journal.pone.0198268)
Supplement: S2 Table — (PDF) [file pone.0198268.s008.pdf]

Cell number in 10000 m^2	P10	P20	P30	P50
MeCP2+ / GABAergic neurons	1.0 ± 0.12 / 1.02 ± 0.12	0.72 ± 0.17 / 0.72 ± 0.17	0.73 ± 0.06 / 0.73 ± 0.06	0.64 ± 0.10 / 0.64 ± 0.10
MeCP2+ / Glutamatergic neurons	1.42 ± 0.37 / 13.23 ± 0.66	7.38 ± 1.79 / 8.82 ± 2.14	7.31 ± 0.34 / 8.24 ± 0.42	7.84 ± 0.31 / 8.26 ± 0.32

S2 Table

MeCP2+ cell number in GABAergic neurons (GAD+,Nissl+) and MeCP2+ cell number in glutamatergic(GAD-,Nissl+) neurons per 10000 μm^2 area during development.
