# Supplementary material for: Experience-dependent MeCP2 expression in the excitatory cells of mouse visual thalamus
Source: PLoS One. 2018 May 30;13(5):e0198268. doi: 10.1371/journal.pone.0198268 (PMC5976183; doi:10.1371/journal.pone.0198268)
Supplement: S3 Table — (PDF) [file pone.0198268.s009.pdf]

	P10	P20	P30	P50
Proportion of GABAergic neurons in the dLGN neurons	7.0 ± 08 %	7.7 ± 0.8 %	8.1 ± 0.6%	7.0 ± 0.9%

S3 Table

Proportion of GABAergic neurons (GAD+, Nissl+) in the dLGN neurons during development.
